# Complete Coding Genome Sequence of a Novel Porcine Reproductive and Respiratory Syndrome Virus 2 Restriction Fragment Length Polymorphism 1-4-4 Lineage 1C Variant Identified in Iowa, USA

**DOI:** 10.1128/MRA.00448-21

**Published:** 2021-05-27

**Authors:** Giovani Trevisan, Ganwu Li, Cesar A. A. Moura, Katie Coleman, Pete Thomas, Jianqiang Zhang, Philip Gauger, Michael Zeller, Daniel Linhares

**Affiliations:** a Veterinary Diagnostic and Production Animal Medicine, Iowa State University, Ames, Iowa, USA; b Iowa Select Farms, Iowa Falls, Iowa, USA; Portland State University

## Abstract

A porcine reproductive and respiratory syndrome virus 2 strain was identified in lung samples from nursery piglets associated with a 17.15% mortality rate on a swine farm in Iowa. Open reading frame 5 (ORF5) sequencing indicated that this strain is a restriction fragment length polymorphism (RFLP) 1-4-4 lineage 1C variant strain, and its complete coding genome sequence was determined.

## ANNOUNCEMENT

Porcine reproductive and respiratory syndrome virus (PRRSV) belongs to the genus *Betaarterivirus*, family *Arteriviridae*, and order *Nidovirales* (1). PRRSV is currently classified into two distinct species, *Betaarterivirus suid 1* (PRRSV-1) and *Betaarterivirus suid 2* (PRRSV-2) (2, 3). Since the concomitant emergence of PRRSV-1 and PRRSV-2 at the end of the 1980s (4–8), PRRSV has spread to most swine-producing countries. In the last quarter of 2020, veterinarians from Iowa and Minnesota, in the Midwest region of the United States, reported the presence of a highly pathogenic PRRSV-2 strain (9, 10) that was later named a PRRSV restriction fragment length polymorphism (RFLP) 1-4-4 lineage 1C (L1C) variant strain based on open reading frame 5 (ORF5) sequence analysis (9, 10). In this study, three piglets were euthanized from a group of nursery pigs demonstrating a high percentage of pigs with thumping, coughing, and mortality, and lung samples were collected. At the end of the nursery phase, the mortality reached 17.50% (2,815 of 16,078). The lung homogenate samples were pooled and tested by reverse transcriptase quantitative PCR (RT-qPCR) (cycle threshold [*C_T_*], 14.9) and PRRSV ORF5 sequencing was performed at the Iowa State University Veterinary Diagnostic Laboratory according to previously described protocols (11). The recovered ORF5 sequence had 99.7% nucleotide identity with strains reported to the SDRS project (9, 12, 13) and identified as an RFLP 1-4-4 L1C variant strain.

The pooled lung homogenate sample was used to determine the whole-genome sequence via next-generation sequencing technology. Total nucleic acid was extracted using the MagMAX pathogen RNA/DNA kit and a KingFisher system (Thermo Fisher Scientific, MA) (11). All software tools were run with default parameters unless otherwise specified. Double-stranded cDNA was synthesized using the NEXTflex rapid transcriptome sequencing (RNA-seq) kit (Bioo Scientific Corp., TX). The sequencing library was prepared using the Nextera XT DNA library preparation kit (Illumina, CA) with dual indexing. The concentration of the sequencing library was determined using a Qubit 2.0 fluorometer (Life Technologies, Carlsbad, CA) and then normalized to a concentration of 2 nM. The normalized library was sequenced on the MiSeq platform (Illumina) using a 2 × 250-cycle MiSeq reagent microkit v2 (Illumina). In total, 1,139,580 raw sequencing reads were preprocessed using Trimmomatic v0.36 to remove adapters and trim the low-quality ends, deleting sequences with a length of less than 36 nucleotides (nt) and using FastQC (14) for sequencing quality analysis. The cleaned reads were classified using Kraken v0.10.5-β (15); 2,684 PRRSV reads (about 28-fold coverage obtained after the genome was assembled) were extracted and *de novo* assembled using SPAdes v3.5.0 as described previously (16, 17).

A near full-length genomic sequence of 15,058 bp, with a GC content of 52.72%, was obtained. A BLASTn search was conducted using the PubMed NCBI library and indicated that the nearly complete genome sequence of this PRRSV-2 RFLP 1-4-4 L1C variant strain shared the highest nucleotide identity (99.6%) with a strain having an incomplete PRRSV genome sequence of 12,949 bp (GenBank accession no. MW646386), recovered in the United States in 2020. The RFLP 1-4-4 L1C variant strain (MW887655) had the next closest nucleotide identity (95.7%) with PRRSV isolate 7705R-S1 (MN073102; 14,952 bp), recovered in the United States in 2018 and belonging to RFLP 1-7-4 L1A.

### Data availability.

The PRRSV whole-genome sequence reported here has been deposited in GenBank under accession no. MW887655. The Illumina MiSeq reads have been deposited in the SRA under BioProject accession no. PRJNA727217.
